# Editorial: Exploring and expanding the protein universe with non-canonical amino acids

**DOI:** 10.3389/fmolb.2023.1303286

**Published:** 2023-10-12

**Authors:** Gustavo Fuertes, Kensaku Sakamoto, Nediljko Budisa

**Affiliations:** ^1^ Laboratory of Biomolecular Recognition, Institute of Biotechnology of the Czech Academy of Sciences, Vestec, Czechia; ^2^ Laboratory for Nonnatural Amino Acid Technology, RIKEN Center for Biosystems Dynamics Research, Yokohama, Japan; ^3^ Chemical Synthetic Biology Group, Department of Chemistry, University of Manitoba, Winnipeg, Canada

**Keywords:** non-canonical amino acid (ncAA), genetic code expansion (GCE), stop codon suppression (SCS), sense codon reassignment (SCR), unnatural base pair (UBP), cell-free translation system (CFTS), aminoacyl tRNA synthetase (aaRS), orthogonal life

In just 30 years, genetic code engineering has allowed us to expand the repertoire of amino acids in proteins from nature’s 20 canonical amino acids to more than 250 non-canonical amino acids (ncAAs), including non-α-amino acids (Fricke et al., 2023), and the trend is increasing. It is now possible to incorporate many types of amino acid substrates (natural and unnatural, long/heavy/bulky, aliphatic, aromatic, halogenated, etc.) into recombinant proteins. Such a field development, especially genetic code expansion (GCE) by orthogonal pairs and expanded genetic alphabet, is unprecedented in natural sciences. Applications of ncAAs are diverse, ranging from biochemical studies of protein-activity relationships with atomic precision to the generation of protein-based polymers with novel functions and ultimately novel life forms. Despite the advantages of ncAAs, the understanding, manipulation, and design of protein structure, dynamics, and function still largely rely on canonical amino acids. Our long-term mission is to transform GCE into a routine toolbox for many laboratories and industries.

The present Research Topic “*Exploring and Expanding the Protein Universe with Non-canonical Amino Acids*” aims to provide the reader with the fundamentals of GCE along with the latest advances. The Research Topic contains 1 review and 8 original research articles, with contributions from both experts and newcomers in the field.

An excellent starting point for anyone interested in genetic code engineering is the review paper by Kimoto and Hirao **,** who discuss both natural base pairs (NBP) and unnatural base pairs (UBP) from the perspective of codon-anticodon interactions. The NBP system includes stop codon suppression (SCS), four-base codon-anticodon interactions, and sense codon reprogramming (SCR). SCS, and in particular, in-frame amber (UAG) codon suppression, is by far the most popular implementation of GCE. In fact, only one original research paper ventures to do SCR, the work by Tittle et al. The authors of this study conclude that in the absence of queuosine nucleoside found in the anticodons of some *E. coli* tRNAs, reassignment of sense codons is slightly enhanced.

Although our Research Topic is dominated by intact cells as platforms for the incorporation of ncAA, cell-free translation systems (CFTS) are also presented. CFTS are particularly useful for expressing so-called “difficult” proteins such as toxins and membrane proteins that would otherwise threaten cell viability. Moreover, the current practice involves encoding the components in plasmids, which may not always be stable. Schloβhauer et al. developed two orthogonal eukaryotic CFTS derived from the Chinese Hamster Ovary (CHO) cell line. The first system is based on transient transfection and expression of aminoacyl-tRNA synthetases (aaRS) prior to cell disruption for extract preparation. The second system is based on stable transfected cells containing aaRS expression cassettes at a defined locus created by the CRISPR/cas9 genomic editing system.

The majority of studies using GCE methods focus on single-point mutations. From a technical perspective, the difficulty of incorporating a particular ncAA at multiple sites is a direct consequence of the relatively low efficiency of most aaRS variants, although the chemical instability of some ncAAs may also play a role. Gueta et al. reported a set of powerful aaRS for the incorporation of 15 different aromatic ncAAs at up to 10 residue positions in the elastin-like polypeptide (ELP), an intrinsically disordered protein. Koch et al. chose a different approach to increase the yield of genetically encoded protein nitration. They prevented the reduction of nitro groups by engineering an *E. coli* strain with reduced nitroreductase activity. The result is an ELP variant carrying up to 60 copies of a nitrobenzyl-containing ncAA, which is the largest number of ncAAs ever introduced in a single polypeptide.

An even greater challenge is the incorporation of two or more distinct ncAAs, partially due to the lack of mutually orthogonal aaRS/tRNA pairs, quintuply orthogonal being the current frontier (Beattie et al., 2023). The two most common aaRS employed in GCE campaigns are the tyrosyl-tRNA synthetase from *Methanocaldococcus jannaschii* (*Mj*TyrRS) and the pyrrolysyl-tRNA synthetase (PylRS) from *Methanosarcinae*. Other PylRS from *Methanomethylophilus alvus* and, more recently, from *Methanococcoides burtonii* (Koch et al., 2023) are also rapidly gaining momentum. Fisher et al. evolved an optimized *Ma*PylRS variant (*Ma*PylRS_opt_) by phage-assisted non-continuous evolution. *Ma*PylRS_opt_ is hyperactive, specifically recognizes Nε-substituted lysines and certain phenylalanine derivatives, but not *para*-substituted ones, and is orthogonal to *Mj*TyrRS, making it an excellent tool for the single and dual incorporation of diverse ncAAs. Another method established by Morosky et al. allows the incorporation of selenocysteine (the 21st proteinogenic amino acid) and Nε-acetyl-lysine (a common post-translational modification) at UGA and UAG codons, respectively. As a result, acetylated selenoproteins can be produced in *E. coli* by dual SCS.

Sometimes, a protein of interest cannot be readily produced in a given host. This is the case with the selenoprotein thioredoxin reductase (trxR1) in mammalian cells. To circumvent this problem, Wright et al. fused a cell-penetrating peptide tag derived from the trans-activator of transcription (TAT) protein of human immunodeficiency virus. Purified TAT-trxR1, recombinantly expressed in *E. coli* by GCE, is efficiently uptaken by human cells, providing a new platform to study such a protein *in situ*.

Finally, another application of ncAAs is in the area of photocontrol to switch/turn protein activity ON and OFF. Pham et al. report the use of a photocaged tyrosine (NBY) to control the binding affinity between two medically relevant proteins, interleukin-24 (a cytokine) and its receptor IL-20R2, by UV light. Cell signaling through the JAK/STAT phosphorylation cascade can, thus, be regulated as needed.

It has been almost three decades since the field of genetic code engineering emerged in the 1990s, and during this time, we have witnessed not only significant advancements in methodology but also the emergence of intriguing concepts. For instance, Szostak and his colleagues proposed that approximately 70% of codons could be reassigned (Herman et al., 2007). Similarly, Söll and his research team estimated that it might be possible to encode the genetic makeup of an organism using only 30 to 40 sense codons (Krishnakumar et al., 2013), leaving over 20 sense codons available for reassignment with ncAAs (Mukai et al., 2015). Against this background, we are pleased that the articles in our Research Topic contribute by presenting various aspects of reprogramming the genetic code, from basic principles to practical examples. These efforts are aimed at fostering further advancements in this technology. For instance, merging orthogonal translation with synthetic metabolism (Völler and Budisa, 2017) would reduce the need for external supplementation of ncAAs (or nucleobases).

Undoubtedly, the unexplored potential of ncAAs will attract researchers from diverse disciplines, including AI, material science, biophysics, biomedicine, and evolutionary biology, among others, to engage in this captivating field. Thanks to these collaborative endeavors, the boundaries of the protein universe and life itself will be pushed, explored, and expanded.
